# Warm Moist Air in Bronchial Inflammation

**Published:** 1846-04

**Authors:** 


*Dr. Golding Bird on warm moist Air in Bronchial Inflammation.*—Were I endeavoring to point out one class of cases more distressing and anxious than another, I would sketch one of pneumonia, or capillary bronchitis, affecting a part of both lungs, and occurring in a child barely emerging from the influence of the poison of measles or hooping cough. The little sufferer, weak from previous disease, tosses restlessly from side to side, with its head thrown back over the pillow or nurse's lap, to straighten the trachea, snatching short and imperfect slumbers, or rather moments of stupor, only to awaken to fresh distress; the livid hue of the finger-nails and toes, the dusky face, parched lips, distended and parting nostrils, the lustrous, prominent eye, present a graphic picture of impending suffocation; whilst the hurried respiration, the catching at the bed-clothes, or throwing the arms over the head, show the efforts made by the child to facilitate the distention of the obstructed lungs. If pneumonia be alone present, all these symptioms, it must be recollected, often exist without a particle of cough; the most severe cases of this kind I have met with, have been absolutely free from it, and have been often mistaken for mere fever. It must never, moreover, be forgotten, that whilst in the adult pneumonia is frequently limited to one lung, in children it usually affects both. The possible absence of violent inflammatory fever, also, must never be forgotten, for ignorance of the fact has often caused the disease to be overlooked. The prominent symptoms of the most dreaded variety, or suffocative pneumonia, are, diminished oxygenation of the blood, and depressed state of the cerebral functions. The little patients are often affected with great somnolence, pale and livid complexion; the glowing heat of surface soon disappears, and is followed by cold and often violet-colored extremities. The respiration is often nearly entirely performed by aid of the abdominal muscles, the action of the diaphragm being violent; a state of things familiar to every physician, and to which attention has of late been especially directed by a German author, Dr. Berg. The distress of the little patient becomes much greater if capillary bronchitis, or inflammation of the lining membrane of the smaller tubes, be superadded; for here, in addition to the consolidation of the hepatized portion of the lung, the very route to the cells is first obstructed by the swollen and congested state of the lining membrane, and afterwards by the exudation of a stiff, viscid, gum-like mucus. And if the larger tubes become involved, as is usually the case after hooping cough, the loud and coarse râle, or hooping sounds, show how great is the effort required to expand the lungs.

In the treatment of these cases, all the therapeutical remedies at our command are too frequently put in requisition in vain; the mercury, antimony, opium and depletion, are all called upon for their assistance; and although, fortunately often with success, still we have sometimes to deplore their inefficiency, and even occasionally to doubt whether, after all, the disease has not been over-treated; and to inquire whether, in efforts to save, the catastrophe has not been rather accelerated than averted.

It is in these cases that I think we possess a most energetic remedial agent, in the shape of an appeal to the functions of the skin and mucous membrane of the air-passages, which, while its effects are always palliative, and often successful, can never be regarded as dangerous. Even the temporary relief afforded by the warm bath, familiar to all practitioners, shows how much is gained by calling upon the skin to do its duty. But even here, too carelessly as this remedy is often used, I think I have seen more ultimate harm than good accrue, by rendering the child more susceptible to the influence of the cold air which it inspires.

In cases of this kind I would urge the practitioner not to consider he has done his best towards saving his little patient, until he has placed the child in an atmosphere, the temperature and moisture of which shall appeal to the skin, and relieve the inflamed tissue. The mode in which I am accustomed to effect this is as follows:

Selecting, if the choice be permitted, a bed-room as small and as free from draughts of air as possible, the windows are carefully closed, and if the casements do not fit accurately, strips of paper should be pasted over the junctures. A stout sheet or blanket should then be fastened with a nail or two to the lintel of the door, outside, so as to hang down and prevent currents of cold air from entering the room during the ingress and egress of the attendants, a large fire being lighted in the grate, which should never be allowed to go out during the treatment. A thermometer should be suspended over the patient's bed, so as to be about two or three feet from its centre, and carefully watched. The indications of this instrument must constitute the sole guide for raising or depressing the fire, and a temperature of from 70° to 78° should be constantly maintained. A large kettle of water is placed on the hob, and kept boiling, so that a current of steam may be constantly poured into the room from its spout, which, for this purpose, must be enlarged by the addition of a few feet of gas-pipe, and until this be procured, with a tube of stiff paper or thin mill-board.

By these precautions the room may, without the slightest difficulty, be kept at a nearly constant temperature for the requisite time. Indeed, it is remarkable how little variation is observed in the thermometric indications, when the most ordinary care is taken to prevent the entrance of long-continued draughts of air. Let us now suppose that a child, the subject of capillary bronchitis or pneumonia, be exposed to the influence of a bed-room arranged with these precautions, placed in bed, and supplied freely with diluents, as tea, toast-water, or common water, for which the little patients generally crave, and inquire what are the probable results of this treatment, independent of any other.—*London Lancet.*

